# Vaginal Microbiome Characterization of Nellore Cattle Using Metagenomic Analysis

**DOI:** 10.1371/journal.pone.0143294

**Published:** 2015-11-24

**Authors:** Mateus Laguardia-Nascimento, Kelly Moreira Grillo Ribeiro Branco, Marcela Ribeiro Gasparini, Silvia Giannattasio-Ferraz, Laura Rabelo Leite, Flávio Marcos Gomes Araujo, Anna Christina de Matos Salim, Jacques Robert Nicoli, Guilherme Corrêa de Oliveira, Edel Figueiredo Barbosa-Stancioli

**Affiliations:** 1 Departmento de Microbiologia, Instituto de Ciencias Biologicas, Universidade Federal Minas Gerais, Belo Horizonte, MG, Brazil; 2 René Rachou Research Center – Fiocruz, Belo Horizonte, MG, Brazil; Cairo University, EGYPT

## Abstract

Understanding of microbial communities inhabiting cattle vaginal tract may lead to a better comprehension of bovine physiology and reproductive health being of great economic interest. Up to date, studies involving cattle microbiota are focused on the gastrointestinal tract, and little is known about the vaginal microbiota. This study aimed to investigate the vaginal microbiome in Nellore cattle, heifers and cows, pregnant and non-pregnant, using a culture independent approach. The main bacterial phyla found were *Firmicutes* (~40–50%), *Bacteroidetes* (~15–25%) and *Proteobacteria* (~5–25%), in addition to ~10–20% of non-classified bacteria. 45–55% of the samples were represented by only ten OTUs: *Aeribacillus*, *Bacteroides*, *Clostridium*, *Ruminococcus*, *Rikenella*, *Alistipes*, *Bacillus*, *Eubacterium*, *Prevotella* and non-classified bacteria. Interestingly, microbiota from all 20 animals could be grouped according to the respiratory metabolism of the main OTUs found, creating three groups of vaginal microbiota in cattle. Archaeal samples were dominated by the *Methanobrevibacter* genus (*Euryarchaeota*, ~55–70%). *Ascomycota* was the main fungal phylum (~80–95%) and *Mycosphaerella* the most abundant genus (~70–85%). Hormonal influence was not clear, but a tendency for the reduction of bacterial and increase of archaeal populations in pregnant animals was observed. Eukaryotes did not vary significantly between pregnant and non-pregnant animals, but tended to be more abundant on cows than on heifers. The present work describes a great microbial variability in the vaginal community among the evaluated animals and groups (heifers and cows, pregnant and non-pregnant), which is significantly different from the findings previously reported using culture dependent methods, pointing out the need for further studies on this issue. The microbiome found also indicates that the vaginal colonization appears to be influenced by the gastrointestinal community.

## Introduction

Cattle ranching has accompanied humankind for thousands of years, and today there are around one billion heads worldwide [1]. These animals are an important part of global economy—constituting a billion dollar market—especially for Brazil, the world’s larger beef exporter [1]. Despite the relevance of these animals, many aspects of their biology are still unknown, including the composition of the vaginal microbiota in cows.

Vaginal microbiota in women shows low species diversity and is dominated by the genus *Lactobacillus*, especially *L*. *crispatus*, *L*. *jensenii*, *L*. *gasseri*, *L*. *johnsoni*, and *L*. *iners* [2–4]. Similar microbiota were described for monkeys and rats [5,6], but the vaginal microbiota in cows remains relatively unknown. Few studies using culture dependent methods reported a low abundant microbiota, dominated by enterobacteria [7–9], but the real microbial composition remains to be defined.

Since the development of next generation sequencing techniques, studies in metagenomics increased widely in number and importance. Today, a number of studies involving the investigation of microbiotas and microbial ecosystems are possible because of the new sequencing platforms, which enabled the generation of massive amounts of information at a low cost per sequenced base. Among the numerous environments explored through metagenomics are the bovine gastrointestinal tract (GIT) [10] and rumen [11,12], and the human gastrointestinal and genitourinary tracts [13], but the bovine vaginal tract (VT) is only starting to be explored [14].

This work describes the vaginal microbiome of Nellore cattle in four different scenarios: non-pregnant heifers, pregnant heifers, non-pregnant cows and pregnant cows.

## Materials and Methods

### Farm, Animals and Sample Collection

In order to investigate the unbiased indigenous vaginal microbiota, an elite herd was selected, composed of pure by origin Nellore cattle (the most important beef cattle in Brazil), with assisted reproduction by insemination. Within the herd, 20 animals that did not present any reproductive clinical signs for the past 12 months were selected, and divided equally in four groups: Non-Pregnant Heifers (NPH), Pregnant Heifers (PH), Non-Pregnant Cows (NPC) and Pregnant Cows (PC). NPH were not older than eighteen months, PH varied between one and two years old and NPC and PC varied between two and five years old. Sample collection took place in May 2013, being the pregnant animals in the first trimester of pregnancy. This study was approved by the Ethics Committee in Animal Experimentation (CETEA/UFMG), approval number 95/2012 and, since it was a private herd, all the procedures were previously approved by the owner.

For sampling, the vulva was washed with distilled water and 70% ethanol, and then 50 ml of sterile saline solution was introduced into the vaginal cavity of the animal, through a syringe coupled to a sterile probe. Then, the vaginal wash was aspirated and kept at 4°C until processing (which occurred on the same day).

### Nucleic acid extraction and amplification

Wash samples were lyophilized to reduce sample volume and total DNA was extracted using DNeasy Blood & Cell Culture DNA Midi Kit (Qiagen, Venlo, Netherlands) according to the manufacturer’s instructions. This DNA was used as template in PCR reactions using universal primers for *Bacteria*, *Archaea* and fungi (Table 1). The reactions were conducted as follows. For *Bacteria*: a mix containing 10 pmol of each primer, 4 μl of 5x buffer (Promega, Fitchburg, Wisconsin, USA), 1 U of GoTaq (Promega), 200 μM of dNTP, 1.5 mM of MgCl_2_, 20 ng of template DNA and nuclease-free water to a final volume of 20 μl was subjected to the following cycling: 95°C for 5 minutes, 30 cycles of 95°C for 40 seconds, 55°C for 40 seconds, 72°C for 1 minute, and finally 72°C for 7 minutes. For *Archaea*: a mix containing 25 pmol of each primer, 45 μl of Taq Platinum Supermix (Thermo Fischer Scientific, Waltham, USA), 80 ng of template DNA and nuclease-free water to a final volume of 50 μl was subjected to the following cycling: 94°C for 4 minutes, 35 cycles of 94°C for 30 seconds, 60°C for 30 seconds, 72°C for 40 seconds and finally 72°C for 10 minutes. For fungi: a mix containing 10 pmol of each primer, 12.5 μl of 2x GoTaq Green Master Mix (Promega), 40 ng of template DNA and nuclease-free water to a final volume of 25 μl was subjected to the following cycling: 95°C for 2 minutes, 35 cycles of 95°C for 15 seconds, 54°C for 25 seconds, 72°C for 20 seconds and finally 72°C for 10 minutes.

**Table 1:** Universal primers used in the PCR reaction for *Bacteria*, *Archaea* and fungi.

**Organism**	***Bacteria***	***Archaea***	**Fungi**
Target region	16S rRNA	16S rRNA	D1/D2
Reference	[15]	[16]	[17]
Primer (Forward)	784F: AGG ATT AGA TAC CCT GGT A	300fEyAr: AGC RRG AGC CCG GAG ATG G	NL1: GCA TAT CAA TAA GCG GAG GAA AAG
Primer (Reverse)	1061R: CRR CAC GAG CTG ACG AC	954rEyAr: CGG CGT TGA RTC CAA TTA AAC	NL4: GGT CCG TGT TTC AAG ACG G
Expected amplicon size	~280bp	~500-600bp	~400–1000bp

### Metagenomic libraries construction and sequencing

Paired-ends libraries were constructed using 50 ng of amplicons obtained in the previously described PCRs. Due to the amplicon size difference between the three targeted biological groups, two different strategies were used. For *Bacteria*, whose amplicon was smaller than 300 bp, DNA fragments were directly coupled to specific adapters using the TruSeq Nano DNA Library Preparation Kit (Illumina, San Diego, USA), according to the manufacturer’s instructions. Next, the DNA-adapter molecules were purified and submitted to amplification reactions using specific primers targeting the adapters. Amplification products were quantified by real time PCR using the SYBR Fast qPCR Kit (Kapa Biosystems, Wilmington, USA). The libraries were then diluted in a Tris-HCl and 0.1% Tween solution, deposited on a flow cell and subjected to 600 sequencing cycles (2 x 300 bp) using the MiSeq Reagent Kit v3 (Illumina).

For *Archaea* and fungi, DNA samples from the same animal were pooled. Then, a similar preparation was performed but, sincesome amplicons were as big as 1000 bp, samples were submitted to a random fragmentation, where DNA was simultaneously fragmented to smaller segments and coupled to specific adapters, using the Nextera XT DNA Library Preparation Kit (Illumina), according to the manufacturer’s instructions. Amplification, quantification and sequencing protocols were performed as for bacterial libraries.

These procedures resulted in the construction and sequencing of two libraries for each animal, one containing bacterial DNA and other containing archaeal and fungal DNA. In order to perform only two sequencing reactions—one for *Bacteria* and one for *Archaea*/Fungi—comprising all 20 animals, samples from the same animal were marked with a distinct index, using the Nextera XT Index Kit (24 indices, 96 samples) (Illumina), according to the manufacturer’s instructions. Thus, it was possible to sequence all 20 animals in the same flow cell and to separate the results according to the index.

### Data processing and analysis

Sequenced paired-ends libraries were submitted to the MG-RAST server [18], where the library pairs where merged based on the homology of the different ends, eliminating those which lacked their respective pair-ends. The merged pairs where then submitted to the server’s pipeline. Sample dereplication was performed in order to remove artificial replicates produced by amplification and sequencing, and sequences were screened for removal of *Bos taurus* sequences, all using the MG-RAST plataform. The dynamic trimming option was selected, aiming the elimination of low quality sequences, requiring a 15 minimal phred score for each base and a limit of five low quality bases per fragment.

The high quality sequences generated were used in the subsequent analysis, which required 98% minimal identity, 100 bp minimal alignment and 10^−8^ maximum E-value for the determination of an Operational Taxonomic Unit (OTU), using MG-RAST. M5RNA database—which comprises Greengenes, RDP and Silva databases—was selected for annotation comparison, being a suitable base for various rRNA sequence analysis. In order to obtain a high fidelity result and diminish the influence of individual variability and extreme values, a maximum of one value per group was excluded in data analysis and graphic construction, after the comparison against the database, when it was too different from the group average, allowing a more realistic overview of the group’s vaginal microbiota. All the results were analyzed on GraphPad Prism 5 by One-Way ANOVA with Tukey’s post-test, with p < 0.05.

## Results and Discussion

### Libraries sequencing data

The rarefaction curves of all 40 sequenced libraries, as well as the sequencing information overview and the quality control filtered sequences for each one can be found in S1 Table and S1 and S2 Figs. Results showed that the sample OTUs were satisfactorily represented for all libraries, since all the curves reached a plateau. These data indicate that the environment was adequately explored, giving a true representation of the OTUs and the OTU diversity present in each sample. Hence, the analysis of such samples should provide an overview of the whole microbiome, not only a fraction of the residing microorganisms.

**Fig 1:**
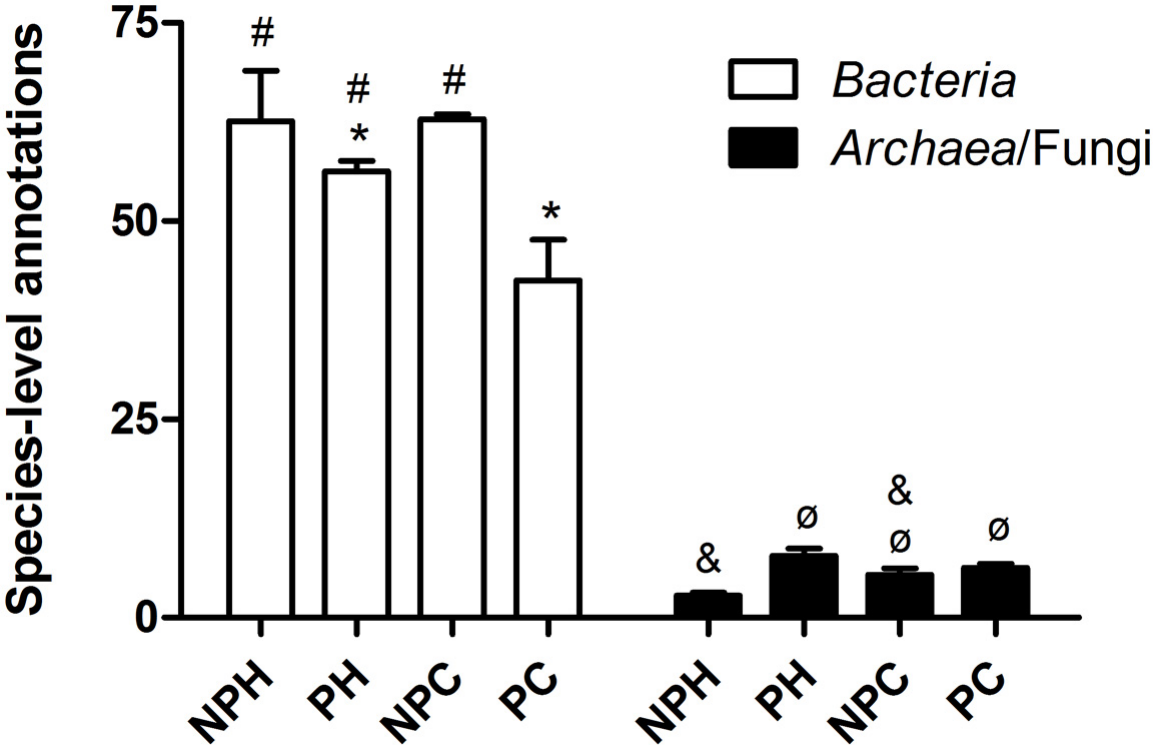
Average alpha diversity in the analyzed groups, expressed as antilog of Shannon’s index. NPH—Non-pregnant Heifers, PH—Pregnant Heifers, NPC—Non-pregnant Cows, PC—Pregnant Cows. Different symbols represent statistical difference (p < 0.05).

Fig 1 represents samples alpha diversity, calculated by the MG-RAST server as the antilog of the Shannon index. This is an important analysis that considers the number of species present in a given sample and their abundance, providing an estimate of the diversity of that sample—a low value of alpha diversity being attributed to communities dominated by only a few species, and a high value to communities dominated by a great variety of members. Bacterial libraries display a much higher alpha diversity index than archaeal/fungal libraries, characterizing a variable environment, while archaeal/fungal microbiota displays a much lower diversity.

Fig 1 also shows that pregnant animals tend to display a lower bacterial alpha diversity when compared to non-pregnant animals, which could be interpreted as a tendency to reduction of bacterial species abundance and/or increase of few organisms abundance during pregnancy. The opposite can be observed for *archaea*/fungi: there was a tendency to an increase in alpha diversity during pregnancy, which can be interpreted as a higher number of species inside the genitourinary tract and/or a lower dominance by the most abundant species.

### Domains

**Fig 2:**
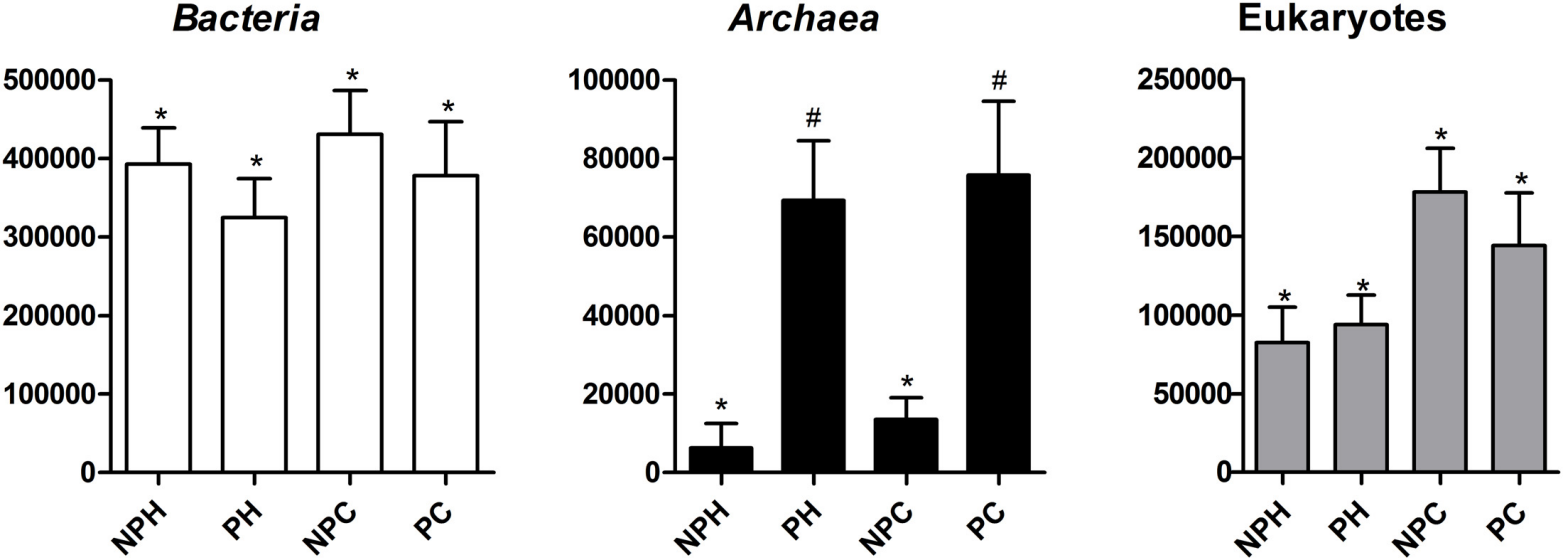
Domains abundance in each group. NPH—Non-pregnant Heifers, PH—Pregnant Heifers, NPC—Non-pregnant Cows, PC—Pregnant Cows. Different symbols represent statistical difference (p < 0.05).

The variability observed within each group (NPH, PH, NPC, PC) was remarkable, as indicated by standard deviations in Fig 2, which presents the absolute abundance of all three biological domains. Although it was possible to observe tendencies when comparing the four groups, in most cases the averages were not statistically different due to the high individual variance within each group. These data suggest a dynamic environment, in which the major components are not static but vary with time, or even the existence of different microbial communities, as has been stated for the human TGI [13].

There was a tendency of reduction on bacterial abundance in pregnant animals, and an increase in *Archaea*, data supported by Fig 2. A probable explanation for such finding is based on the work of Otero and colleagues [7]: These authors described the variation of cultured microbial population during the estrous cycle in cattle, and found a tendency for reduction of the microbial population after estrus (stage marked by high levels of estrogen). Thus, in the period dominated by progesterone release, the animals probably tend to present a less abundant bacterial microbiota. Since progesterone is the dominant hormone throughout most of pregnancy, vaginal bacterial microbiota could behave this way, exhibiting a slight reduction in pregnant females. After parturition, and the return to normal estrous cycle, microbial population in the vagina would return to the equilibrium prior to pregnancy, with increase in bacterial and reduction in archaeal populations.

Variation of archaeal population was significant (p < 0.05) between pregnant and non-pregnant animals of similar age (NPH x PH and NPC x PC), but no statistical difference was observed in NPH x NPC and PH x PC comparisons. This suggests that the momentary hormonal state has more influence on the vaginal archaeal population than age and hormonal maturity.

Since few non-fungal eukaryote reads were present in our samples, the domain analysis shown displays all observed eukaryotes. As expected—since the PCR was designed for fungi—this domain was dominated by fungi, hence posterior analysis are presented as fungal phyla and genera, although the relative abundance of the fungal OTUs refers to the total eukaryote reads found. Data presented in Fig 2 show a tendency of increase in eukaryotes number in cows. These results may be related to morphological changes brought by hormonal and physical maturity of these animals, or a possible colonization of this tract by eukaryotic organisms throughout the cattle lifecycle, since these organisms do not appear to be influenced by the different bovine hormones levels and classes, at least not in their population numbers.

### Phyla and genera analysis

#### Bacteria

**Fig 3:**
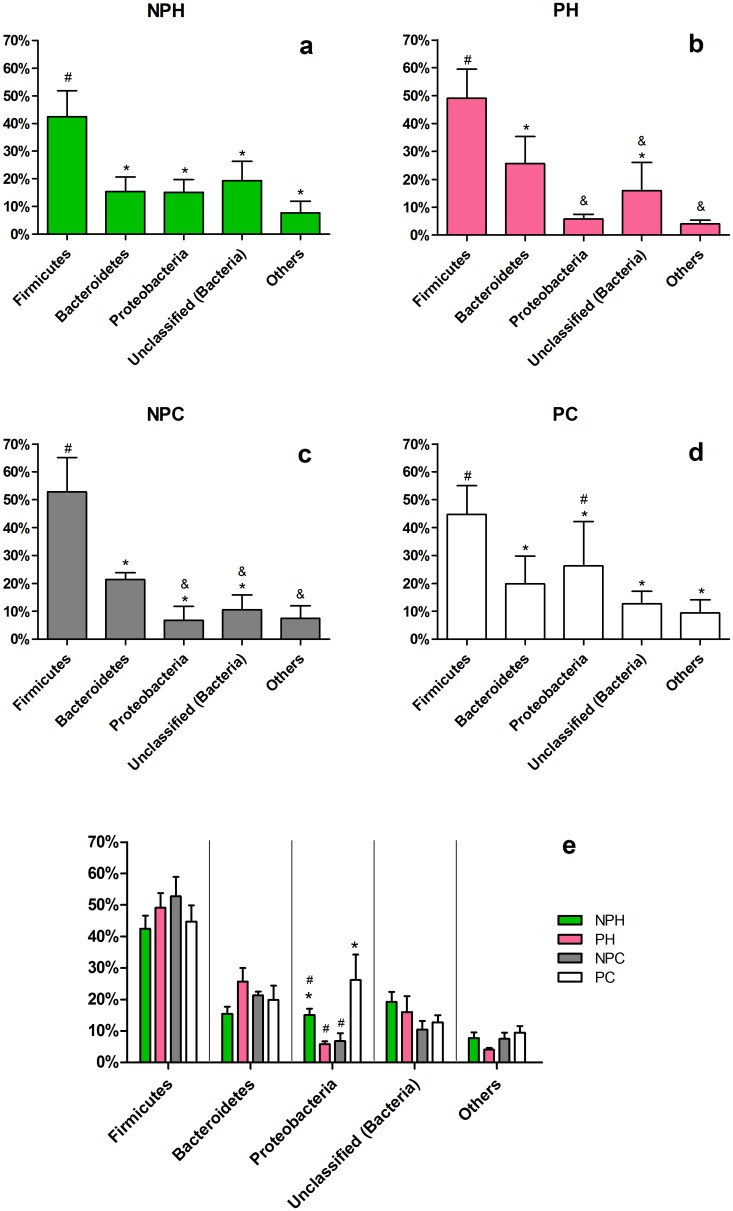
Bacterial phyla found in the vaginal tract. Relative abundance comparisons within each group are shown in graphics *a* to *d*, and phyla comparison between groups are shown in graphic *e*. NPH—Non-pregnant Heifers, PH—Pregnant Heifers, NPC—Non-pregnant Cows, PC—Pregnant Cows. Different symbols represent statistical difference (p < 0.05).

Bacterial phyla found are listed in Fig 3. The three most abundant phyla inside Nellore VT were *Firmicutes*, *Bacteroidetes* and *Proteobacteria*. Malmuthuge and colleagues [19], also using metagenomics, found the same three dominant phyla in the gastrointestinal microbiota of three weeks old cattle, data corroborated by the works of Ziemer [20] and Kim [21], studying feces cultures from different animal species, also through metagenomics. In the present study *Firmicutes* appeared as the most abundant phylum (p < 0.05) in all groups (in pregnant cows it was the most abundant either, but without statistical difference). Relative abundance of *Proteobacteria* in pregnant cows showed a very high variation (more than 50%), making difficult any analysis.

**Fig 4:**
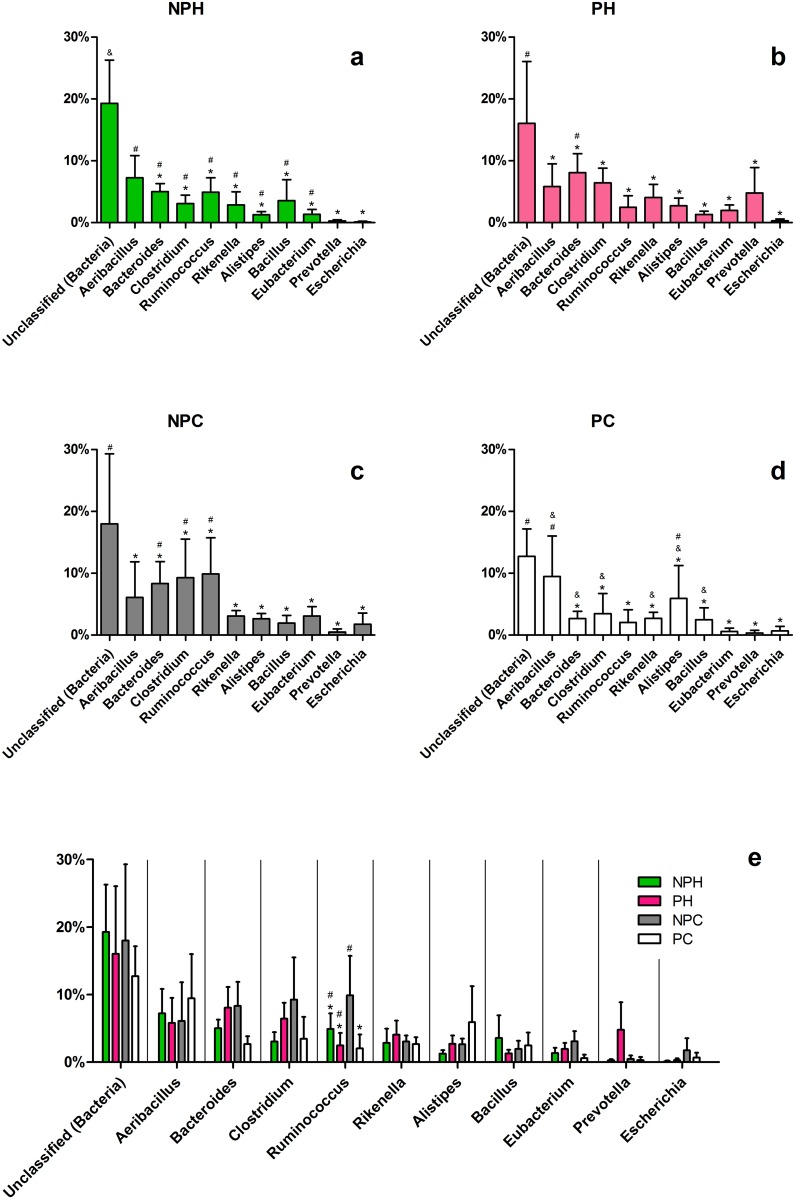
Major bacterial OTUs at the genus-level in each group. Average relative abundance is presented for the ten most abundant bacterial OTUs in the vaginal tract. (*a-d*) Comparison within each group. (e) Comparison between groups. NPH—Non-pregnant Heifers, PH—Pregnant Heifers, NPC—Non-pregnant Cows, PC—Pregnant Cows. Different symbols represent statistical difference (p < 0.05).

**Fig 5:**
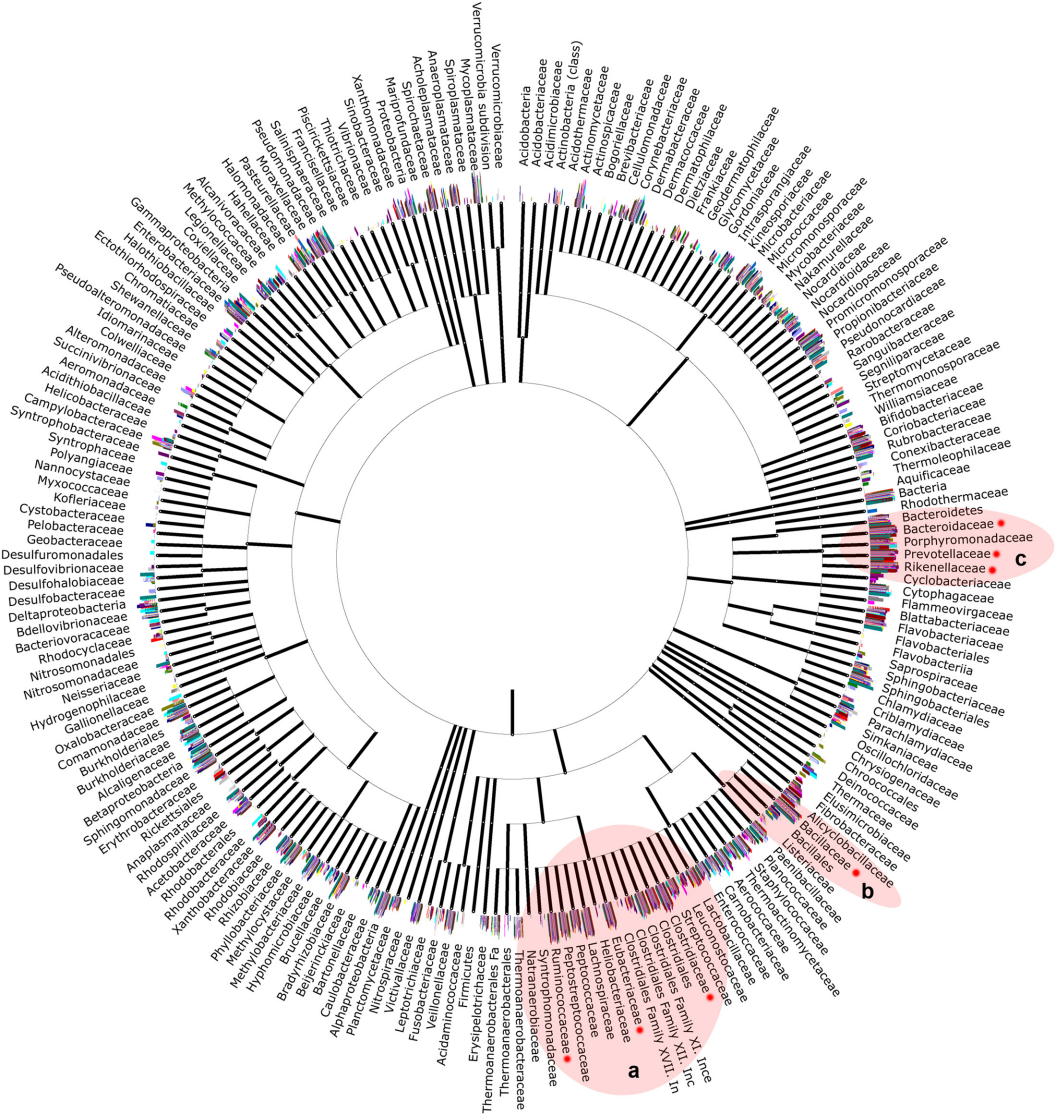
Bacterial phylogenetic tree, including all bacterial families encountered in the samples. The seven major families, comprising nine of the ten most abundant OTUs at the genus level are indicated by the red dots. Red circles indicate three important orders, namely *Clostridiales* (a), *Bacillales* (b) and *Bacteroidales* (c).

In a more detailed analysis, a wide variety of bacterial genera seems to colonize the vaginal tract in these animals (Fig 4). The most abundant genera (*Aeribacillus*, *Bacteroides*, *Clostridium*, *Ruminococcus*, *Rikenella*, *Alistipes*, *Bacillus*, *Eubacterium* and *Prevotella*) belong to three different orders—*Clostridiales*, *Bacillales* and *Bacteroidales* (Fig 5)—and comprise 30–40% of total bacteria present in the community.

At the genus level, considering the ten most abundant bacterial OTUs (Fig 4), unclassified *Bacteria* were the major OTU. This finding is not unusual in studies in metagenomics or non-cultured organisms. Several authors have reported the presence of large numbers of unclassified sequences in their work [22–25], a reflection of the vast yet unexplored microorganism universe. In the other nine most abundant OTUs, there was a clear relation between the vaginal microbiota and known bovine GIT microbiota. Eight OTUs have been found in some portion of bovine GIT or feces: *Bacteroides* [19,20,26,27], *Clostridium* [21,26–28], *Ruminococcus* [26,27,29], *Rikenella* [30], *Alistipes* [26], *Bacillus* [31], *Eubacterium* [27,31,32] and *Prevotella* [12,21,26,27]. Interestingly, the only genre that has not yet been related to bovine GIT, or GIT of any other animal species, was the most abundant: *Aeribacillus* (*Firmicutes*). This genus was first described in 2010, differentiated from *Geobacillus* [33] and is known to house several thermophilic bacteria [33,34]. Recent studies have found these bacteria in the microbiota of some animals, such as fish [35], and more recently they have been described in bovine milk samples [36]. This study now reports, for the first time, a great abundance of this genus in bovine vaginal tract, in all studied groups, emphasizing the importance of this bacterium also in this animal microbiome. *Bacillus* has also been reported in bovine milk, where it can sporulate and survive to pasteurization process, endangering animal production and human health [37]. These two genera found in bovine milk are also the only aerobic ones pertaining to the ten most abundant OTUs in bovine vaginal tract. *Escherichia* was added to this analysis because previous works have reported the isolation of this genus in bovine vaginal samples, as well as other enterobacteria [7,8]. Our analysis shows that these bacteria are not among the major OTUs found, and its dominance in studies involving cultivation can probably be explain by their rapid growth and low nutritional requirements, coupled with an insufficient knowledge of the vaginal microbiota.

A clear dominance could not be found within genera, although *Aeribacillus*, *Bacteroides*, *Clostridium* and *Ruminococcus* appear to be the most abundant in all four groups. Interestingly, it was not possible to associate a specific vaginal microbiota composition with cows/heifers or pregnancy status.

**Fig 6:**
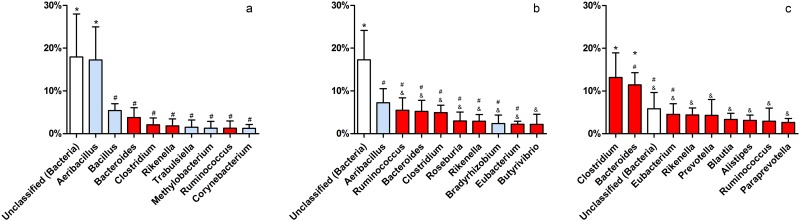
Relative abundance of the 10 major OTUs found at the genus-level in vaginal microbiota types “A” (a), “B” (b) and “C” (c). The blue bars refer to aerobes and facultative anaerobes, red bars to obligate anaerobes and white bars to unspecified bacterial metabolisms. Results are shown as the mean and standard deviation of each group, and different symbols represent statistical difference (p < 0.05).

Analyzing each animal individually, although the aforementioned variability is evident, a pattern can be observed, separating all 20 animals in three distinct groups according to the abundance of the major OTUs found (S3 Fig). The first, group “A”, displays higher *Aeribacillus* and *Bacillus* abundance, and is composed by the animals NPH3, NPH4, PH3, NPC4, PC1, PC3 and PC4. These animals probably present higher amounts of oxygen inside the vaginal cavity, which would explain the abundance of the aerobes *Aeribacillus* and *Bacillus*. When the most abundant OTUs of only these seven animals are analyzed, there are, in fact, five aerotolerant (aerobic/facultative anaerobic) and four obligate anaerobic genera, along with the Unclassified derived from Bacteria (Fig 6). Group “B”, composed by NPH1, NPH2, NPH5, PH2, PH4, NPC5 and PC2, probably presents lower oxygen levels in the vagina, since only two aerotolerant genera are found among the ten most abundant OTUs of this group (Fig 6), although *Aeribacillus* remains as one of the major genera. Finally, group “C”, composed by PH1, PH5, NPC1, NPC3 and PC5, displays only obligate anaerobes among its 10 most abundant OTUs, along with the Unclassified genera (Fig 6). The vaginal cavity of the animals in this group should present minimum amounts of oxygen, limiting or even inhibiting the growth of aerobic species that thrive in groups A and B.

These data indicate that individual variation is more important than age (heifer x cows) or pregnancy status (pregnant x non-pregnant). If there were no statistical differences between NPH, PH, NPC and PC regarding the most abundant bacterial OTUs found at the genus level, individual analysis allowed the identification of three distinct microbial profiles inside the vaginal cavity of these animals, which could not be related to our grouping parameters. In fact, the difference between these three groups could not be related to any parameter analyzed in this study: race/genetics, geographic location of the farm, handling, collection date and method, genetic material treatment, data sequencing and analysis were all the same for all 20 animals. Only individual characteristics remain as an explanation for this variation. Small anatomical, immunological and physiological differences may influence vaginal microbial population even more than any of the characteristics targeted in this study. Similar findings have been reported by authors studying the human microbiome through metagenomics. These studies have identified great individual variation that could only be related to individual characteristics, and proposed the classification of the human GIT microbiota in different enterotypes [13,38]. In a similar fashion, our data suggest that bovine vaginal microbiota can be classified in three different groups, based on individual characteristics, mainly a possible difference in oxygen availability inside the vaginal tract.

One finding is evident in this three-group analysis: No NPH can be found in group C. This group is composed manly by obligate anaerobes, and could be a final stage of microbial community development for animals with low oxygen level in the vaginal tract. If this is true, it is possible that NPHs—which are the youngest animals in this work—present a still developing vaginal microbiota. To this date, no study has demonstrated the dynamics for microbial communities’ development inside the bovine vaginal tract and our results indicates that microbiota maturity for this tract could take more than two years for these animals.

#### Archaea

**Fig 7:**
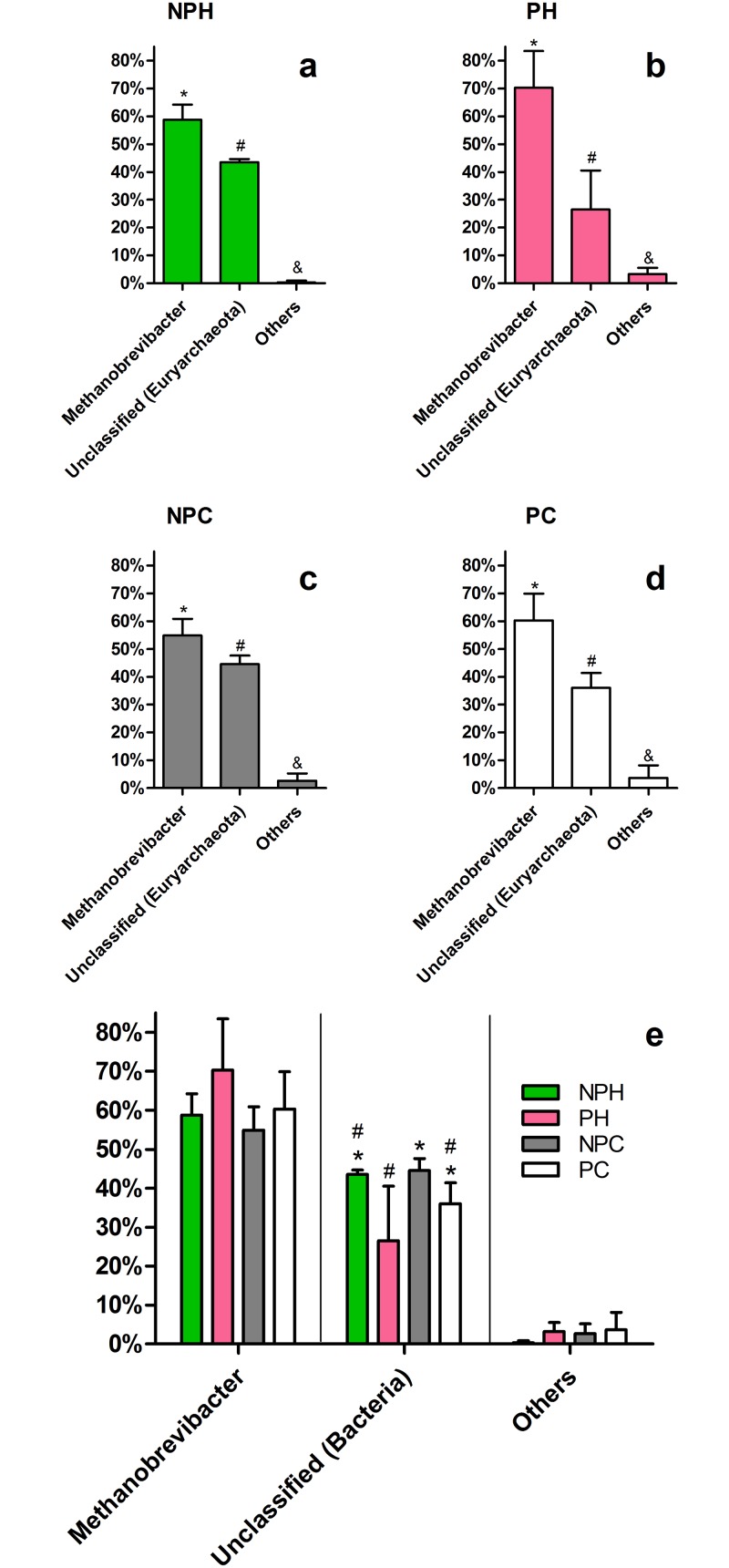
Major archaeal OTUs at the genus-level in each group. Average relative abundance is presented. (*a-d*) Comparison within each group. (e) Comparison between groups. NPH—Non-pregnant Heifers, PH—Pregnant Heifers, NPC—Non-pregnant Cows, PC—Pregnant Cows. Different symbols represent statistical difference (p < 0.05).

Only one archaeal phylum was observed in all four groups, *Euryarchaeota*. Among the genera found, a high prevalence of *Methanobrevibacter* (Fig 7) was observed, representing almost 60% of all *Archaea* in each group. This genus is one of the most important organisms in bovine rumen—having a central role in the metabolism of plant compounds [27,39,40]—and is also present in the GIT of other animals, including humans [41–43]. Individual analysis confirms a great relative abundance of *Methanobrevibacter* in each animal, and a significant number of Unclassified *Euryarchaeota* (S4 Fig). These data again indicate a close relation between vaginal and GIT microbiota in cattle, probably due to the anatomical characteristics of these animals. The exposition of vaginal lumen to large quantities of *Methanobrevibacter* from the GI could explain the origin of this genus inside the vaginal tract. This archeal genus also showed variation in abundance depending on the host hormonal status. Pregnant animals tend to have a higher proportion of *Methanobrevibacter*, rather than of unclassified *Euryarchaeota* (Fig 7), and a similar observation can be made by analyzing the global *Archaea* present in the bovine vaginal tract. Fig 2 shows that *archaeal* populations were significantly more abundant in pregnant than in non-pregnant animals. It is possible that they do not have a marked and important role in normal healthy microbiota, appearing only in a specific context (pregnancy).

#### Eukaryotes

**Fig 8:**
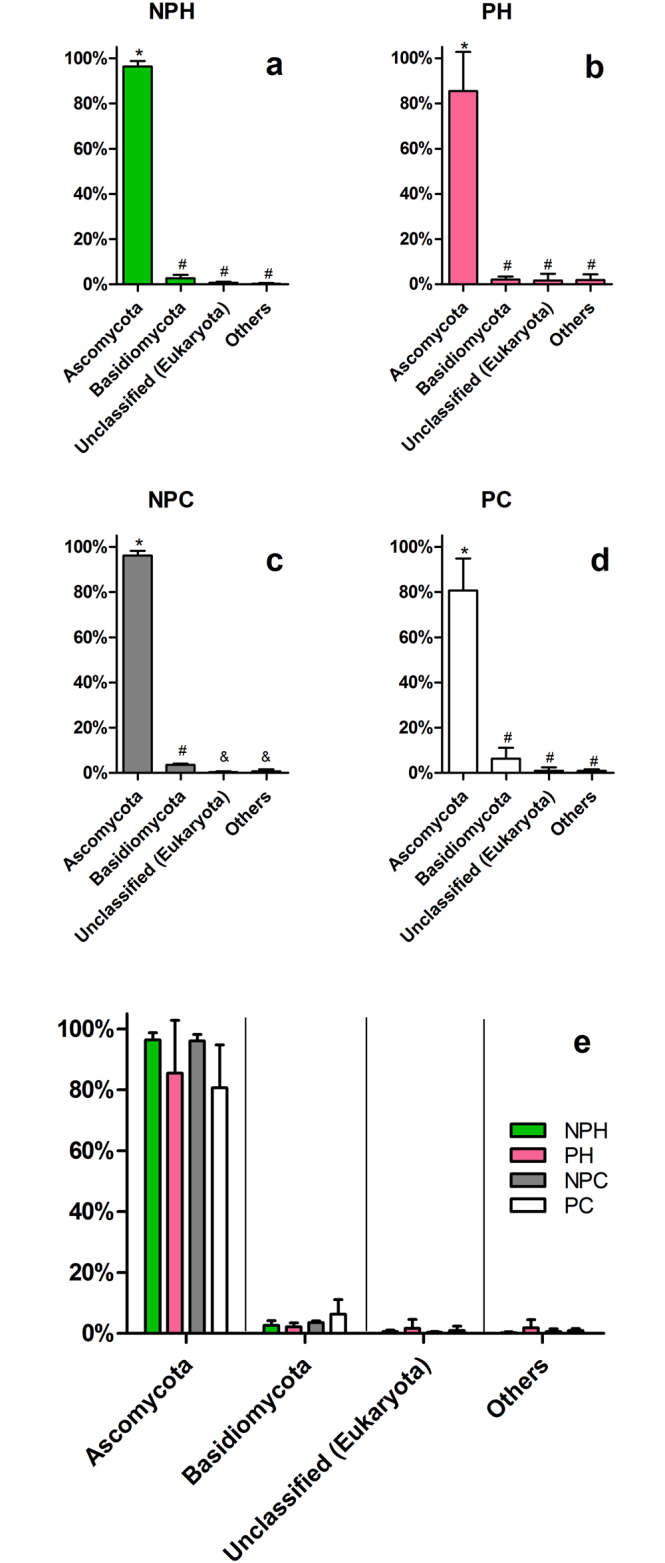
Major eukaryotic phyla found in the vaginal tract. Relative abundance comparisons within each group are shown in graphics *a* to *d*, and phyla comparison between groups are shown in graphic *e*. NPH—Non-pregnant Heifers, PH—Pregnant Heifers, NPC—Non-pregnant Cows, PC—Pregnant Cows. Different symbols represent statistical difference (p < 0.05).

Eukaryote analysis was focused on the presence of fungi, with only two phyla detected (*Ascomycota* and *Basidiomycota*), comprising almost 90% of eukaryotes present in all groups (Fig 8). Ascomycota dominates all groups, showing a tendency to reduction in pregnancy condition (Fig 8).

**Fig 9:**
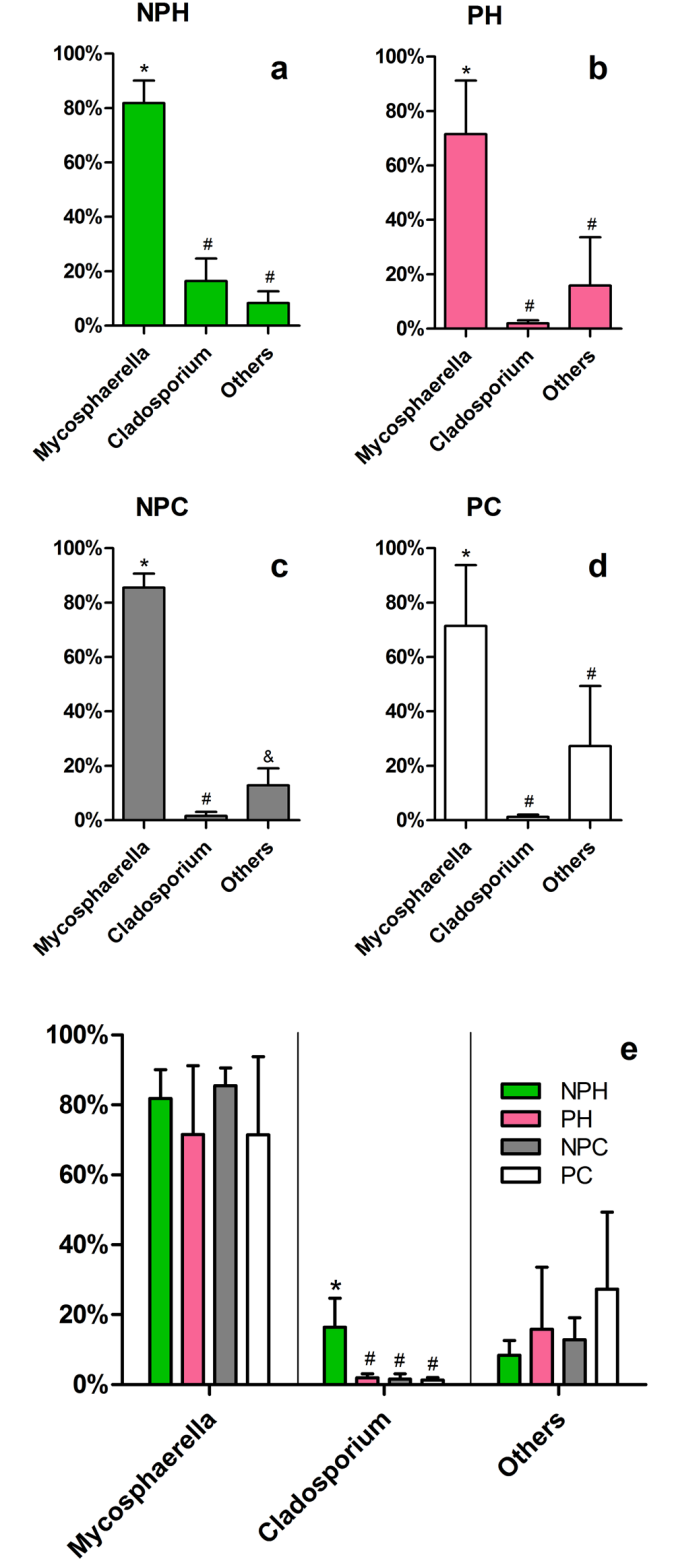
Major eukaryotic OTUs at the genus-level in each group. Average relative abundance is presented. (*a-d*) Comparison within each group. (e) Comparison between groups. NPH—Non-pregnant Heifers, PH—Pregnant Heifers, NPC—Non-pregnant Cows, PC—Pregnant Cows. Different symbols represent statistical difference (p < 0.05).

Among the most abundant eukaryotic OTUs, *Mycosphaerella* genus displays a relative abundance ranging from 70 to 85% in the all groups (Fig 9), dominating over all others, which is also clear in the individual analysis (S5 Fig). Only three animals do not present *Mycosphaerella* as the most abundant OTU, two of them displaying a great number of *Cladosporium* (NPH2 and NPC1), and one dominated by *Phaeosphaeria* (PC2) (S5 Fig). *Mycosphaerella* is an endophytic fungus genus commonly found in soil [44], which houses a few species of grass and other plants pathogens [45,46]. Some authors have reported the efficiency of these fungi in producing compounds with antagonist activity against protozoa [47] and even against other pathogenic fungi [48]. However, to date, no study had described the isolation or even the identification of this organism in an animal microbiota, and this work is the first to report it. Antagonist activity could explain the dominance of this OTU over other fungal genera, allowing this organism to thrive in the vaginal environment, at the same time as it provides protection against pathogens. If bovine vaginal colonization is truly influenced by the GIT, it is likely that the presence of *Mycosphaerella* inside cattle vagina is related to the ingestion of these fungi. Therefore, the presence or absence of this OTU in other animals may strongly depend on handling and environmental factors. Fig 9 also shows a tendency to a decrease in *Mycosphaerella* relative abundance in pregnant animals and to an increase in underrepresented OTUs. This phenomenon, allowing the proliferation of different little represented OTUs is in accordance to what is expected in an immunosuppression context, as is the case for pregnant animals [49,50].

### The GIT x VT relation in cows

All findings presented in this work suggest a strong relationship between the microbiota inhabiting the reproductive and digestive tracts of cattle, which can be explained by their anatomical proximity. In cows, the vulva is located immediately below the anus and is often found covered with feces. The wide opening of the vulva could allow colonization by microorganisms from the digestive tract still in early stages of life. In fact, many diseases of the reproductive tract are caused by microorganisms found in the feces of these animals [51–53], and their presence in vaginal microbiota could explain why the occurrence of postpartum endometritis is so common, occurring in almost 90% of parturient cows [8]. The relationship between feces and the genital tract is so strong that cows with horizontal vulva (which forms an angle >45° with the vertical plane) may accumulate feces inside the vaginal cavity [54]. Urovagina, a medical condition in which urine is pooled in the cranial portion of the vagina, often producing a solution of continuity across this organ, has been positively associated with higher endometritis likelihood [54].

Cows presenting these anatomical abnormalities may have permanent urovagina, but many healthy cows presents it as transient condition immediately after birth, or during estrus [55]. This can have a significant impact on vaginal colonization of adult cows and even heifers, since estrus may happen before the animal reaches puberty [56].

### Formation and maintenance of vaginal microbiota in cattle

Considering all data presented here, as well as the factors involving the vaginal and digestive tracts of cattle, the hypothesis of intestinal origin of the vaginal microbiota seems the most plausible one. Heifers and cows investigated here showed a strong trend in this direction. *Bacteria*, *Archaea* and fungi present in the GIT, habitants or accidentals, could colonize the vagina, eventually forming the microbiota here described. In time, a wide range of organisms directly or indirectly related to the GIT would thrive inside this tract, especially *Mycosphaerella*, *Aeribacillus*, *Bacteroides*, *Clostridium* and *Ruminococcus*, depending on the vaginal microbiota type, in addition to several other also significant organisms present in smaller quantities, having *Methanobrevibacter* as the dominant *Archaea* during pregnancy.

Regarding the bacterial vaginal microbiota, anatomical variations possibly direct vaginal colonization, creating an environment that could favor aerotolerant or obligate anaerobic organisms, or maintain an atmosphere that allows the colonization by bacteria with any of these two metabolisms. It is noteworthy that no NPH showed an anaerobe dominated vaginal microbiota, indicating that these animals have yet to achieve full microbial development.

The results obtained in the present work contrast with the hypothesis of some authors that hormonal maturity would have a significant effect on the formation of vaginal microbiota in cattle [9]. This effect was not observed and cannot be inferred from the data presented. In contrast, our results indicate that individual variation has far more influence on vaginal microbiota than any of the factors investigated in the present study. However, sexual contact could still have an effect in this tract and the microbial community of male genitourinary tract was not investigated. Reproduction in the selected farm was conducted only through artificial insemination, which prevents prediction on the influence of this event on the vaginal microbiota, and future studies should focus on this investigation.

## Conclusion

The present study aimed to characterize the vaginal microbiota in Nellore, the major beef cattle in Brazil, investigating heifers and cows pregnant or non-pregnant. To date, only one other study has exploited the vaginal microbiome using a culture-independent approach: Swartz and colleagues [14] also found great bacterial diversity, and contrasting results with culture-dependent methods, but depicted many different dominant genera from what is presented in this study. This could be related to the different 16S rRNA target used (V3-V4, while we used V5-V6), cattle breed (crossbred x pure Nellore) or even diet and handling, since we believe this tract is directly influenced by the GIT. These findings only confirm the need to further explore this important microbiome. In this study, metagenomic analysis showed great diversity for bacterial microbiota and low diversity for the archaeal and fungal components. The vaginal tract was dominated by a wide variety of *Bacteria*, with a tendency to higher abundance of this domain in non-pregnant animals. Investigations at phylum and genus levels did not show a clear variation between heifers and cows nor between pregnant and non-pregnant animals regarding the dominant OTUs, but established a paradigm on the bacterial communities found inside the bovine vaginal tract, which can be divided in at least three different types, based on the major OTUs found and their metabolism. The most abundant bacterial phyla were *Firmicutes*, *Bacteroidetes* and *Proteobacteria*, while the dominant genera were distributed along many groups, especially *Aeribacillus*, *Bacteroides*, *Clostridium* and *Ruminoccocus*. *Archaea* were present in higher quantities in pregnant animals, having low abundance in the non-pregnant ones. Concerning *Archaea*, the only phylum encountered was *Euryarchaeota*, and the major genus. The eukaryotes tend to be present in higher quantities in older animals (cows), and did not vary significantly with hormonal status, although there was a tendency to a decrease in abundance of dominant OTUs in pregnant animals. *Ascomycota* was the dominant phylum, and *Mycosphaerella* was the major genus in all four studied groups. The results showed a microbiota whose development is strongly influenced by the proximity of the gastrointestinal tract, without displaying a relevant hormonal influence on the microbial ecology, during either puberty or pregnancy. Although many questions raised by our work requires additional studies, which is natural for a pioneer investigation, a solid overview can be identified based on the findings presented here. Future work should involve other bovine races and different handling, immunity and hormonal status in order to confirm our findings, or establish a new paradigm.

## Supporting Information

S1 Fig Rarefaction curves of all 20 bacterial libraries. (TIF)

S2 Fig Rarefaction curves of all 19 archaeal/fungal libraries. (TIF)

S3 Fig Major bacterial OTUs analyzed in each animal. (TIF)

S4 Fig Major archaeal OTUs analyzed in each animal. (TIF)

S5 Fig Major fungal OTUs analyzed in each animal. (TIF)

S1 Table Library and quality control filter analysis of the generated sequences. (DOCX)
