# Supplementary figures and images for: Vaginal Microbiome Characterization of Nellore Cattle Using Metagenomic Analysis
Source: PLoS One. 2015 Nov 24;10(11):e0143294. doi: 10.1371/journal.pone.0143294 (PMC4657983; doi:10.1371/journal.pone.0143294)

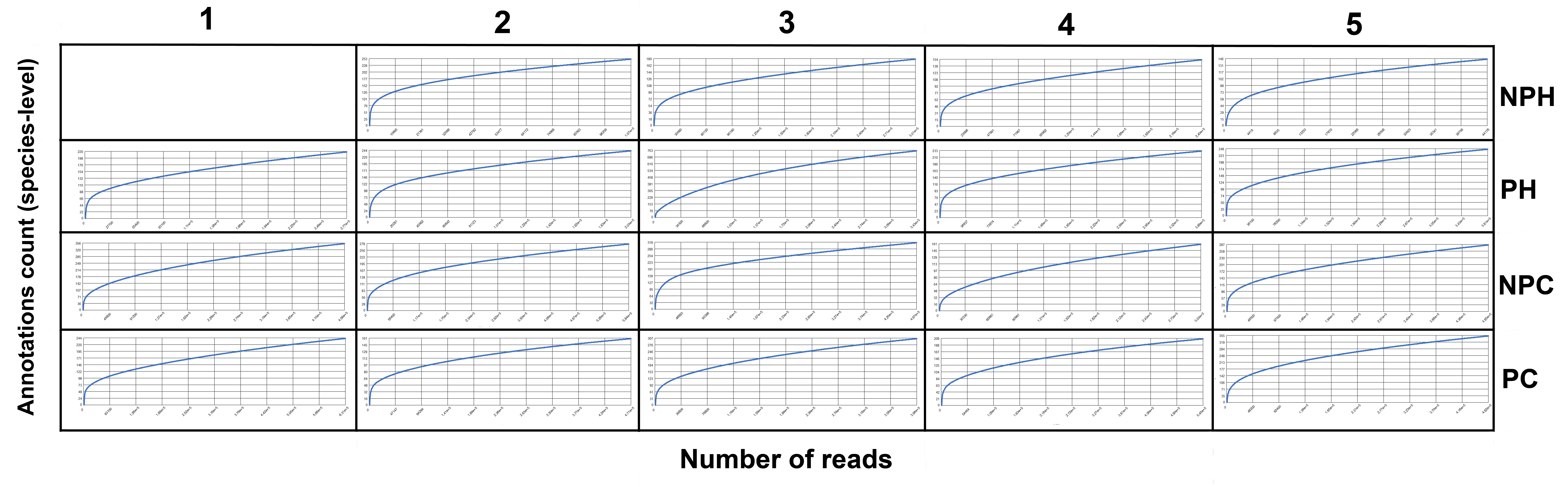

Supplement: S1 Fig — (TIF) [file pone.0143294.s001.tif]

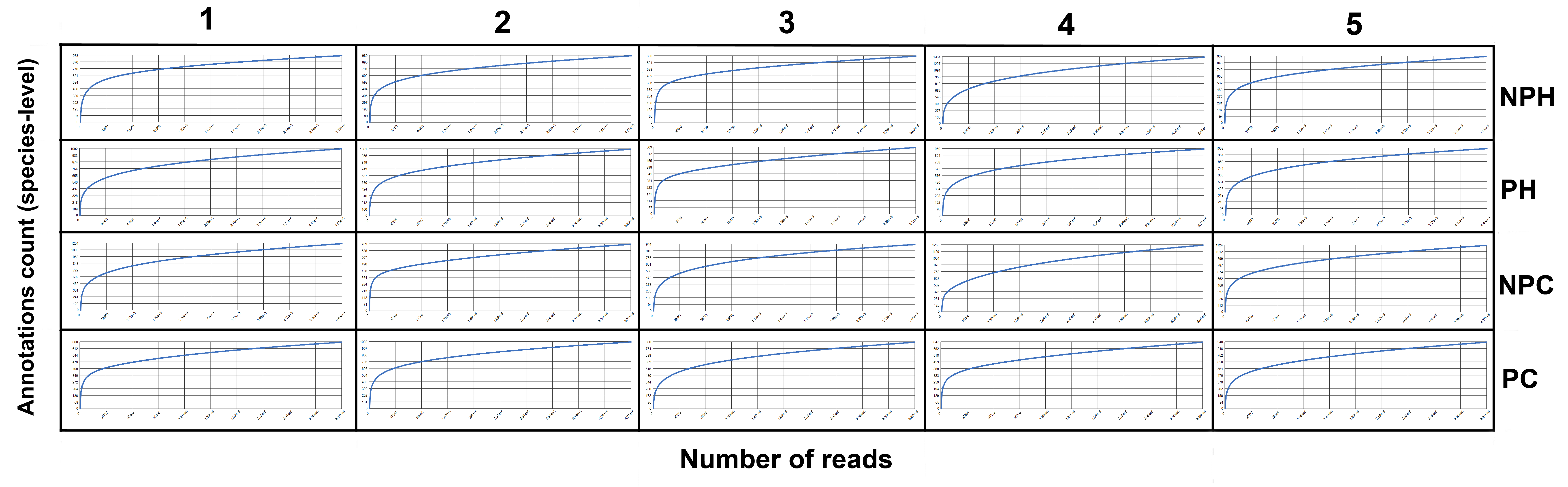

Supplement: S2 Fig — (TIF) [file pone.0143294.s002.tif]

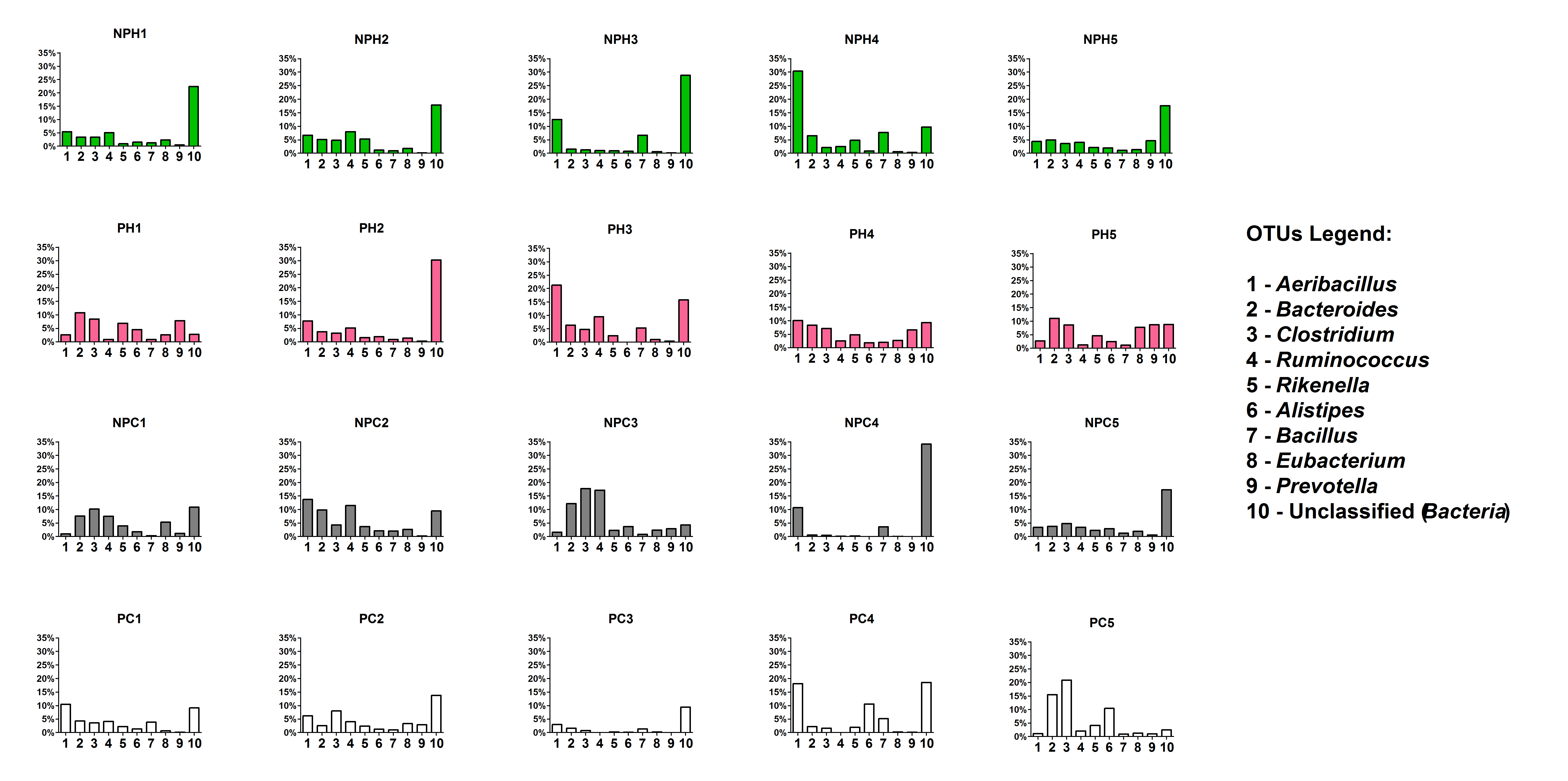

Supplement: S3 Fig — (TIF) [file pone.0143294.s003.tif]

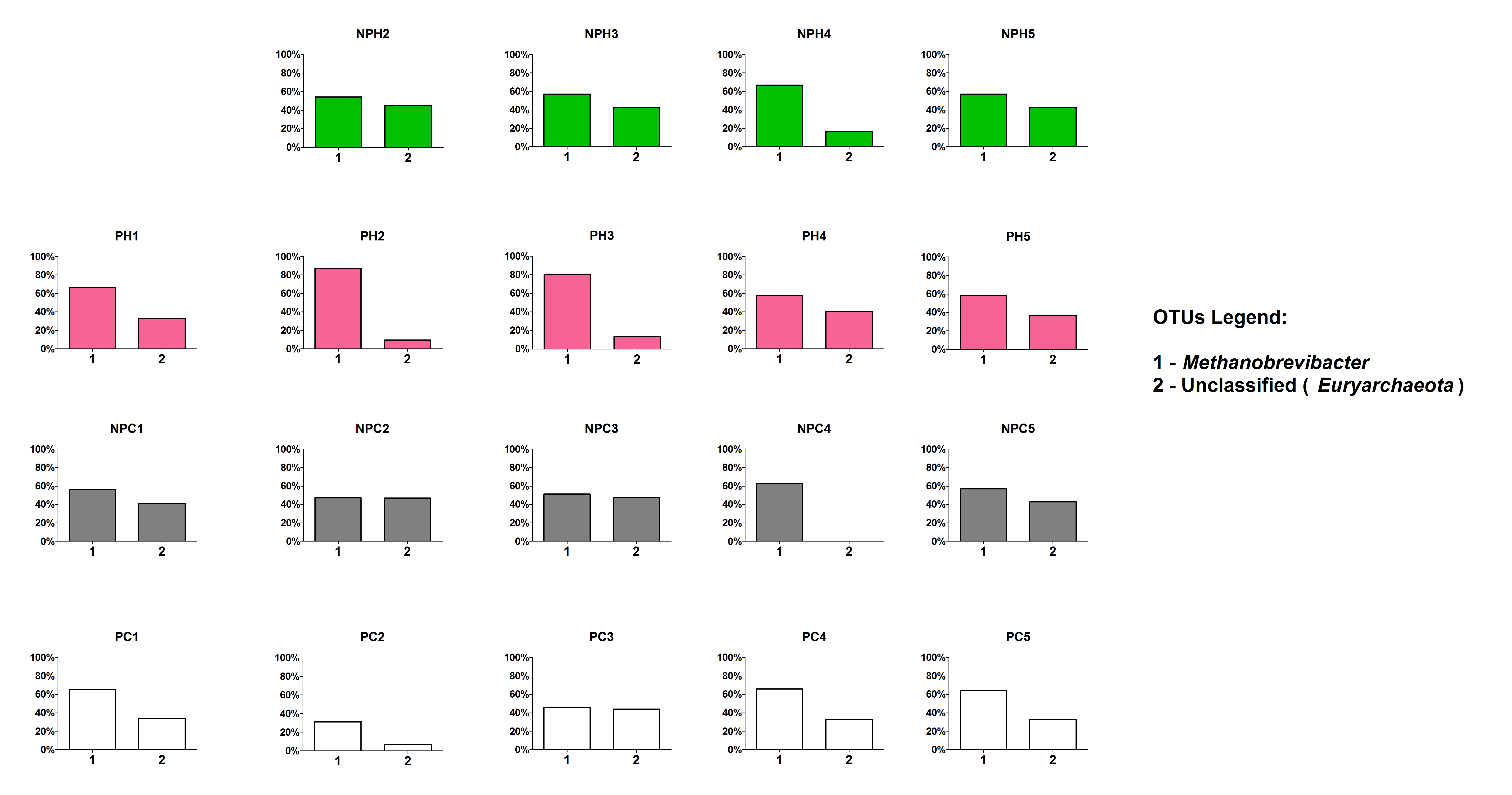

Supplement: S4 Fig — (TIF) [file pone.0143294.s004.tif]

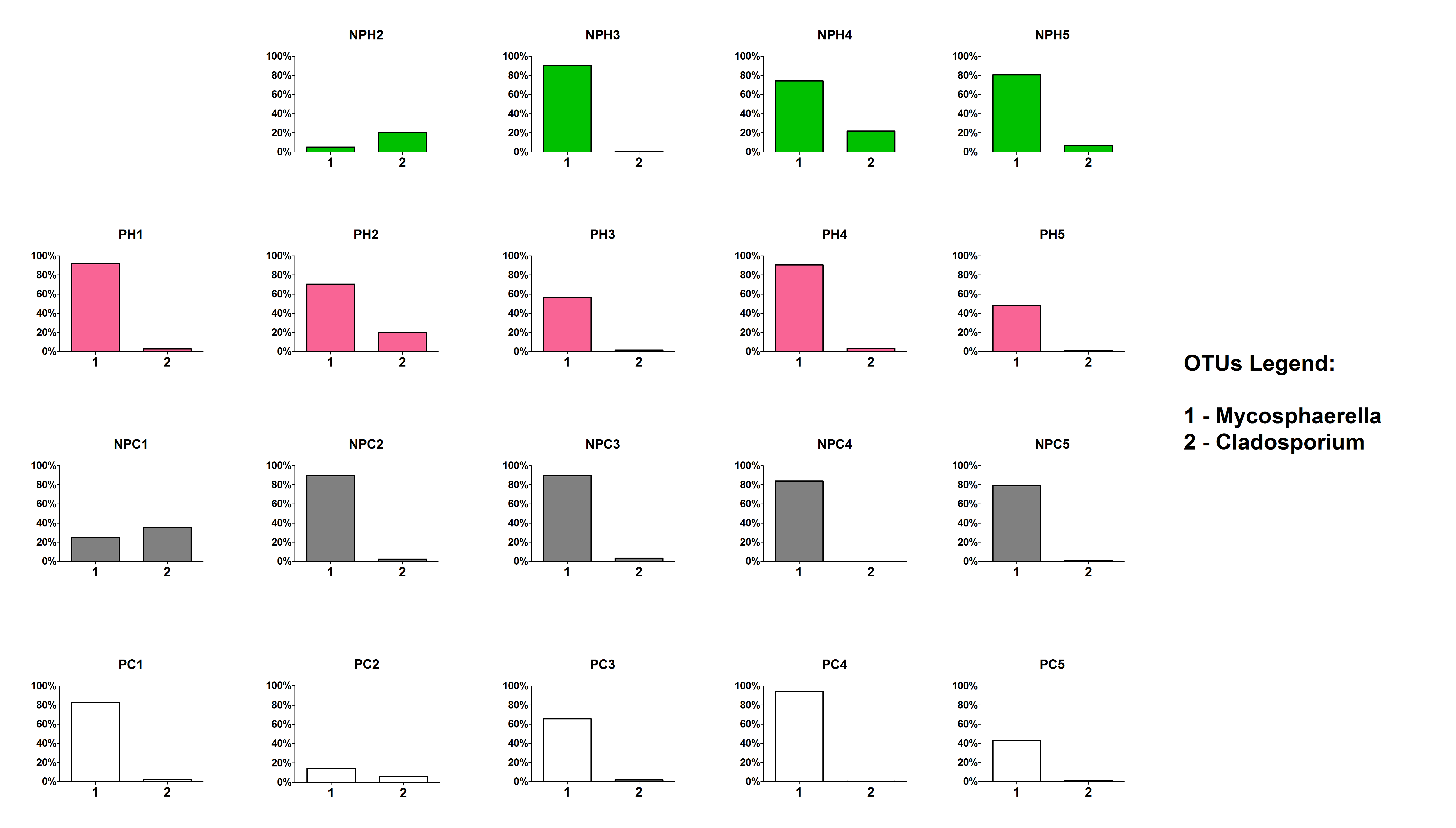

Supplement: S5 Fig — (TIF) [file pone.0143294.s005.tif]
