# Supplementary material for: Vaginal Microbiome Characterization of Nellore Cattle Using Metagenomic Analysis
Source: PLoS One. 2015 Nov 24;10(11):e0143294. doi: 10.1371/journal.pone.0143294 (PMC4657983; doi:10.1371/journal.pone.0143294)
Supplement: S1 Table — (DOCX) [file pone.0143294.s006.docx]

| **Animal** | **Bacterial Sequences** | | | | | **Archaeal/Fungal Sequences** | | | | |
| --- | --- | --- | --- | --- | --- | --- | --- | --- | --- | --- |
|  | Total Sequences | Artifical Replicates Removed | QC Filtered Sequences | QC Passed Sequences | Mean Sequence Length | Total Sequences | Artifical Replicates Removed | QC Filtered Sequences | QC Passed Sequences | Mean Sequence Length |
|  |  |  |  |  |  |  |  |  |  |  |
| **NPH1** | 305058 | 215408 | 15002 | 74648 | 235 ± 67 | - | | | | |
| **NPH2** | 401399 | 303274 | 3985 | 94140 | 232 ± 68 | 106992 | 78215 | 9145 | 19632 | 246 ± 105 |
| **NPH3** | 308810 | 190368 | 50737 | 67705 | 232 ± 71 | 300855 | 254898 | 7312 | 38645 | 208 ± 90 |
| **NPH4** | 544504 | 435737 | 4324 | 104443 | 205 ± 68 | 239998 | 203150 | 7181 | 29667 | 194 ± 91 |
| **NPH5** | 376581 | 240799 | 42492 | 93290 | 223 ± 72 | 44209 | 32137 | 2504 | 9568 | 227 ± 93 |
| **PH1** | 465055 | 364869 | 840 | 99346 | 234 ± 65 | 277130 | 231573 | 14406 | 31151 | 229 ± 101 |
| **PH2** | 368948 | 277755 | 6801 | 84392 | 238 ± 68 | 202971 | 155763 | 17913 | 29295 | 226 ± 101 |
| **PH3** | 251414 | 112481 | 76710 | 62223 | 228 ± 79 | 343260 | 252088 | 52808 | 38364 | 261 ± 110 |
| **PH4** | 326702 | 221385 | 22007 | 83310 | 225 ± 72 | 369559 | 300823 | 21041 | 47695 | 216 ± 99 |
| **PH5** | 446517 | 350657 | 1083 | 94777 | 240 ± 65 | 381001 | 298529 | 40912 | 41560 | 247 ± 114 |
| **NPC1** | 565357 | 446515 | 1508 | 117334 | 232 ± 67 | 456323 | 356624 | 46052 | 53647 | 202 ± 95 |
| **NPC2** | 371053 | 284135 | 10368 | 76550 | 240 ± 69 | 584183 | 487348 | 33313 | 63522 | 209 ± 96 |
| **NPC3** | 283798 | 210737 | 512 | 72549 | 233 ± 69 | 466945 | 401023 | 10616 | 55306 | 210 ± 98 |
| **NPC4** | 661416 | 530261 | 44830 | 86325 | 300 ± 92 | 303353 | 252321 | 28100 | 22932 | 264 ± 116 |
| **NPC5** | 437093 | 341103 | 2957 | 93033 | 234 ± 69 | 485333 | 407732 | 26252 | 51349 | 197 ± 113 |
| **PC1** | 317322 | 235497 | 18777 | 63048 | 239 ± 66 | 631012 | 507182 | 63502 | 60328 | 155 ± 95 |
| **PC2** | 473479 | 358341 | 11731 | 103407 | 240 ± 65 | 471810 | 416762 | 19481 | 35567 | 257 ± 115 |
| **PC3** | 366743 | 312917 | 506 | 53320 | 229 ± 68 | 398153 | 307232 | 40614 | 50307 | 223 ± 104 |
| **PC4** | 322749 | 199497 | 60340 | 62912 | 255 ± 62 | 544704 | 411645 | 81153 | 51906 | 155 ± 94 |
| **PC5** | 360967 | 269778 | 7770 | 83419 | 227 ± 69 | 462356 | 339272 | 72564 | 50520 | 196 ± 109 |

Table S1 – Library and quality control filter analysis of the generated sequences. Samples were filtered to remove artificial replicates, host (*Bos taurus*) sequences, and allow a maximum of five bases with quality score below 15 for each sequence.
